# Prevalence of *Shigella* species and its drug resistance pattern in Ethiopia: a systematic review and meta-analysis

**DOI:** 10.1186/s12941-019-0321-1

**Published:** 2019-07-09

**Authors:** Siraj Hussen, Getamesay Mulatu, Zemenu Yohannes Kassa

**Affiliations:** 1 Department of Medical Laboratory Science, College of Medicine and Health Sciences, Hawassa University, Hawassa, Ethiopia; 2 School of Nursing and Midwifery, College of Medicine and Health Sciences, Hawassa University, Hawassa, Ethiopia

**Keywords:** Systematic review, Meta-analysis, Prevalence, *Shigella* species, Resistance, Ethiopia

## Abstract

**Background:**

*Shigella* species are a major cause of dysentery and may attribute for death worldwide. Currently antibiotic resistance became the critical challenges for management of infectious disease. The aim was to conduct a systematic review and meta-analysis of *Shigella* species and its drug resistance pattern in Ethiopia.

**Methods:**

A comprehensive literature search was conducted through internet searches using database of MEDLINE, PubMed, Google scholar, EMBASE, HINARI, Cochrane Library and reference lists of previous prevalence studies from January 1999 to November 2018. Results were presented in forest plot, tables and figures with 95% CI. The Cochrane Q test and I^2^ test statistic were used to test heterogeneity across studies. The Pooled estimate of *Shigella* species and its drug resistance pattern was computed by a random effects model.

**Results:**

The pooled prevalence of *Shigella* species in Ethiopia was 6.6% (95% CI 4.7–8.8). In the subgroup analysis, the highest prevalence was observed among patients in Health facility (8.5%, 95% CI 6.2–11.5) whereas the lowest prevalence was observed in Community based studies (1.6%, 95% CI 0.8–3.4). In addition, *Shigella* species were highly resistant to ampicillin, amoxicillin, erythromycin and multi-drug resistant (MDR) with the pooled resistance proportions of 83.1% (95% CI 75.7–88.6), 84.1% (95% CI 75.6–90.1), 86.5% (95% CI 70.9–94.4) and 83.2% (95% CI 77.1–87.9), respectively. On the other hand, comparably low resistance pattern was reported for ciprofloxacin 8.9% (95% CI 6.0–12.8), ceftriaxone 9.3% (95% CI 3.9–20.5), and norfloxacin 8.2% (95% CI 3.8–16.6) and gentamycin 17.3% (95% CI 11.2–25.9). Subgroup analyses indicated that study years were associated with a decreasing *Shigella* prevalence over time (p = 0.002).

**Conclusion:**

The pooled estimate showed high burden of *Shigella* infection and its high proportion of drug resistance pattern to ampicillin, amoxicillin and erythromycin in Ethiopia. Therefore, initiating and scale up of performing drug susceptibility test for each shigellosis case, educate the community and health care providers on appropriate use of antibiotics need to be considered and strengthened.

**Electronic supplementary material:**

The online version of this article (10.1186/s12941-019-0321-1) contains supplementary material, which is available to authorized users.

## Background

*Shigella* species are a major cause of dysentery disease and may attribute for death worldwide. Shigellosis is a major public health threat in developing countries like Ethiopia, people living with poor sanitation and overcrowded condition. Annual number of Shigellosis episodes globally estimated to be about 165 million, more than half (100 million) of episodes occurs in low and middle income countries, and attributed for more than 1 million deaths per year. Shigellosis is more prevalent among under five children, and causes dysentery and under five mortality [1, 2].

Antibiotic resistance becomes a critical public health problem around the globe in recent years. Based on 2016 WHO report, *Shigella* species is one of the eight dangerous drug resistance bacteria. Worldwide, there are 700,000 deaths as a result of antimicrobial resistance (AMR) every year. The experts suggest that this figure will rise to 4.2 million in Africa and 10 million globally by 2050, if nothing is done [3–5].

The treatment of Shigellosis has currently become more challenging due to the emergence of drug resistant species and associated with a variety of biological, pharmacological and societal variables with the worst combinations in low and middle income countries [6–8]. Multidrug-resistant *Shigella* significantly vary from area to area of the world in relation with the practice of widespread use of antimicrobial agents [3, 9].

Antibiotic resistance is a natural phenomenon that occurs whenever antibiotics are in use. However, there are human behaviors that contribute to the rapid development and spread of bacterial antibiotic resistance. Availability and use of broad spectrum antibiotic without prescriptions facilitate the development of resistance by *Shigella* species [10].

Different studies have been carried out in different parts of Ethiopia at different times to document the epidemiology of and drug Susceptibility pattern of *Shigella* species. However, there is no summarized prevalence data of this bacterial infection and its drug Susceptibility pattern at country level to help in the formulation of appropriate intervention methods. Therefore, the present study is the first of its kind and aimed to determine the pooled prevalence pooled prevalence and drug susceptibility pattern of *Shigella* species in Ethiopia.

## Methods

### Search strategy

A comprehensive literature search was conducted on the prevalence of *shigella* species and its antimicrobial resistance pattern among Ethiopia population. Potentially relevant studies were identified through a literature search of Medline, PubMed, Google scholar, HINARI and Cochrane Library. The search was based on the combination of the following special index search terms (medical subject headings (MeSH) and Boolean operations: “*Shigella*” AND “Prevalence “OR “Epidemiology” AND “Drug Resistance, Microbial” AND “Dysentery, Bacillary/epidemiology” AND “Ethiopia” “title and abstract” from January 1st 1999 to November 20th 2018. Articles search were focused on published studies with epidemiological and/or clinical data. All records were managed in Endnote version X7 (Clarivate Analytics, Philadelphia, PA, USA). The search was carried out from April 5th, 2016 to November 20, 2018. The limit of language was English and the limit of study group was human.

#### Eligibility criteria

We reviewed abstracts from initial search using defined inclusion and exclusion criteria.

##### Inclusion criteria

Studies were selected for systematic review and meta-analysis, 1, if they were conducted in Ethiopia 2, study design been cross-sectional 3, studies reported the prevalence of *Shigella* species and its drug resistance pattern 4, literatures published in the English language and 5. Published articles were considered.

##### Exclusion criteria

Studies, which were not fully accessed after reading the titles and abstracts were excluded since we are unable to assess the quality of each article in the absence of their full texts.

## Data extraction

The data extraction was done by three researchers (S.H, Z.Y and G.M) independently from included studies using a standardized and pretested format prepared in Microsoft Excel. The data abstraction format included first author, study design, region in the country (study site in the country), publication year, sample size, population characteristics, age group of study participants, prevalence of *Shigella* species, medical treatment type, and resistance pattern of *Shigella* species. Disagreement on data extractions between researchers were resolved through discussion and consensus.

### Quality assessment

The quality was assessed using 9 point Joanna Briggs Institute (JBI) critical appraisal tools, the following criteria is established: sample frame appropriate to address the target population, study participants sampled in an appropriate way, adequate sample size, study participants sampled in an appropriate way, study subjects and the setting described in detail, data analysis conducted with sufficient coverage of the identified sample, valid methods used for the identification of the condition, the condition measured in a standard and reliable way for all participants, appropriate statistical analysis and adequate response rate. Individual studies were assigned a score that was computed using different parameters in line with the review objectives. The responses were scored 0 for “No and not reported” and 1 for “Yes”. Total scores ranged between 0 and 9. Studies with medium (fulfilling 50% of quality assessment parameter) and high quality were included for analysis [11] (Additional file 1: Table S1).

### Statistical analysis

The data entry and analysis were done using Comprehensive Meta-Analysis (version 3.1) software. The original articles were described using forest plot, figures and tables. Since there was heterogeneity among studies, random effect model was used to compute the pooled prevalence and antimicrobial susceptibility of *Shigella* species. The estimated pooled prevalence rate with 95% confidence interval (CI) was presented.

### Sub-group analysis

Sub-group analysis was performed based on Region; (Amhara, Oromia Southern Ethiopia, Central Ethiopia, Hareri and Tigray), age group; (children, adult and all age group), study population (health facility based and community based) and year of study; (1999–2003, 2004–2008, 2009–2013 and 2014–2018).

### Heterogeneity and publication bias

Statistical heterogeneity was evaluated by Cochran’s Q test and I^2^ statistic. The I^2^ provides an estimate of the percentage of the variability in effect estimates that is due to heterogeneity rather than sampling error or chance differences. So, the existence of heterogeneity was verified using Cochran’s Q test (p < 0.10 indicates statistically significant heterogeneity) and I^2^ test that measures level of statistical heterogeneity between studies. I^2^ (values of 25%, 50% and 75% are considered to represent low, medium and high heterogeneity respectively) [12, 13]. Begg intercept and Mazumdar rank correlation statistics test methods were used to statistically assess publication bias (p < 0.05 was consider as suggestive of statistically significant publication bias) [14]. As the results of the test suggested a possible existence of a significant publication bias, the final effect size was determined by applying Duval and Tweedie’s Trim and Fill analysis in the random-effects model.

## Results

### Identified studies

**Fig. 1:**
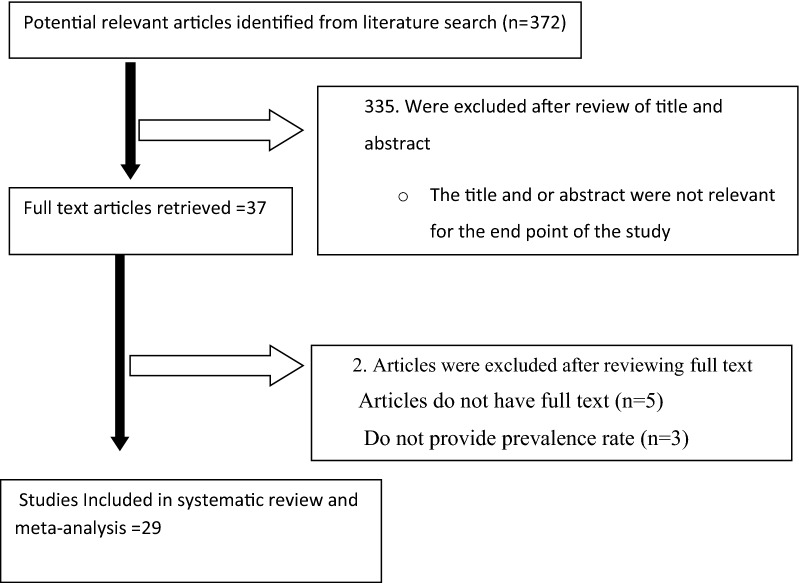
Flow diagram of studies reviewed, screened and included

**Fig. 2:**
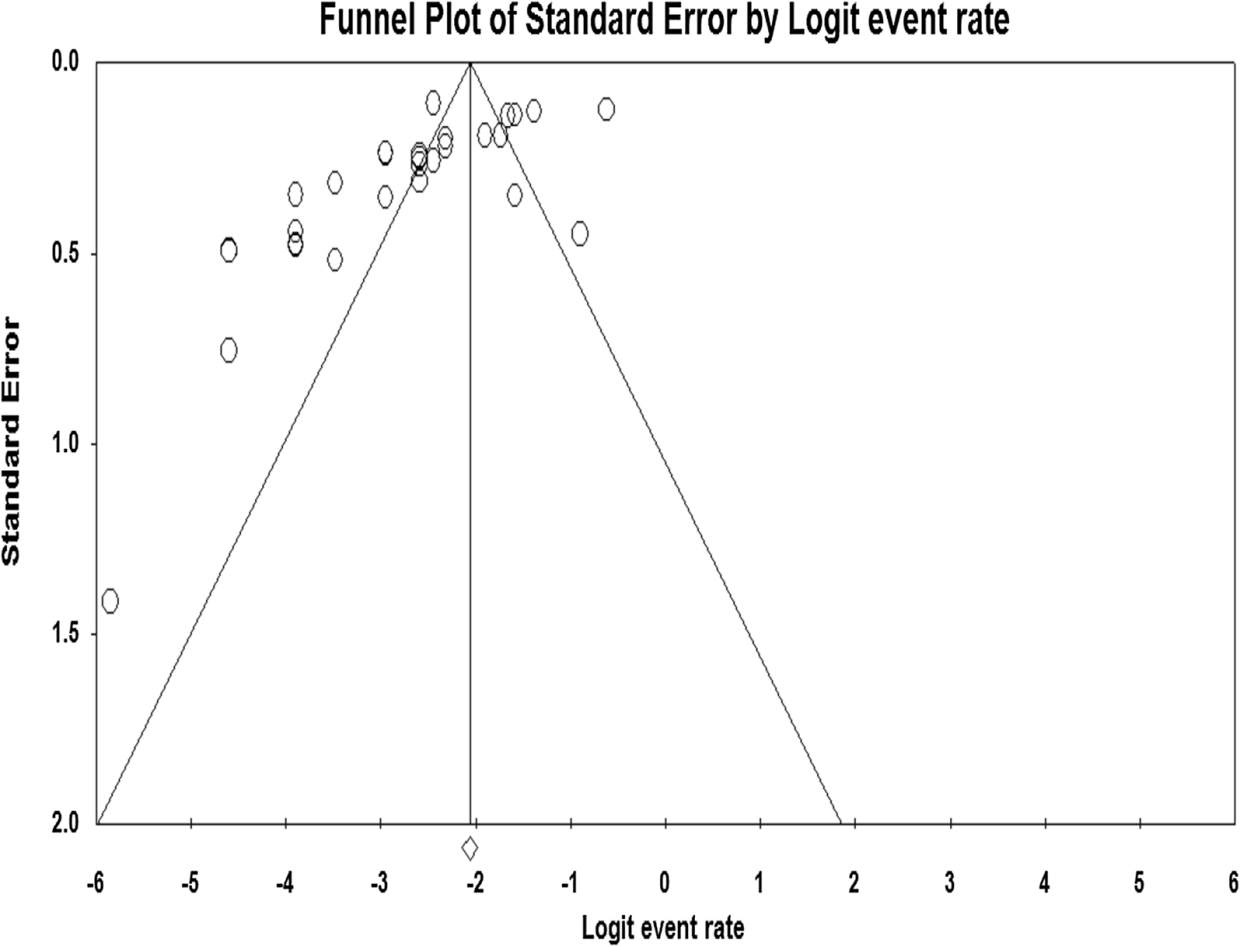
Funnel plot for the prevalence of *Shigella* species in Ethiopia

Through electronic database search, we have found a total of 372 studies. Of which, 335 were excluded based on the titles and abstracts, three article were not reported prevalence of *Shigella* species and the remaining five were excluded due to unavailability of full text articles. Finally, 29 studies were found to be eligible and included in the meta–analysis (Fig. 1). Included articles exhibited high heterogeneity according to Cochrane Q test (Q = 437.836 test p < 0.0001) and I^2^ test (I^2^ = 93.605%), which is indicative to use random effects model. The distribution of the studies using funnel plot (Fig. 2) showed asymmetrical distribution of effect estimate and Eggers regression intercept test (p < 0.0001) and Begg and Mazumdar rank correlation (p < 0.001) indicated evidence of publication bias (Additional file 2: Figures S1 and S2). Therefore, we were used Trim and Fill analysis to adjust the final pooled estimate.

### Study characteristics

Selected articles were published from 1999 to 2018. Also, all included publications were obtained from 5 regions and 1 City administration, but no data was obtained from other regions (Afar, Benishangul-Gumuz, Gambela and Somali). The total study populations involved for the estimation of *Shigella* species were (8521). Of this, 7571 study participants were included in the meta-analysis of *Shigella* species drug resistant pattern. A total of twenty-nine studies were considered eligible for quantitative syntheses. Twenty-five studies were used to estimate the proportions of drug resistant for *Shigella* species (Table 2). Nine studies were used to estimate the prevalence of *Shigella* species in children and seven studies were used to estimate for adult population. Of the total studies, only seven reported the serogroup of *Shigella* (Table 1). Among study populations, 3907 (45.85%) were examined for *Shigella* and its antimicrobial resistance from Amhara region. About 1683 (19.75%) from Oromia region, 1545 (18.13%) from southern region, 425 (4.99%) from Central Ethiopia (Addis Ababa), 244 (2.86%) from Harare region and 717 (8.41%) were from Tigray region. Cross sectional study design was used in all studies. The study population varied from 24 to 1200, and were conducted between the years 1999–2018 [15–43] (Table 1). All studies utilized stool specimens for diagnosis of *Shigella* species (Table 1). Data from health facility based study: 6717 patients with diarrhea and 113 without diarrhea and from community based study: 1691 apparently healthy subjects were considered for quantitative syntheses. The prevalence of *Shigella* in stool samples of apparently healthy subjects and patients ranged from 0.0 to 3.1% from 1.1 to 34.6%, respectively. MacConkey, xylose lysine desoxycholate and Salmonella *Shigella* agar used for culture and disk diffusion method for antimicrobial susptablity (Additional file 1: Table S2).

**Table 1:** Summary of 29 studies reporting the prevalence of *Shigella* in different parts of Ethiopia, from 1999 to 2018

Region	Author and refs.	Pub. year	Study area	Age group	Study base	Sample size	Prevalence (%)	Specimen	Diagnostic methods	Quality score (9 point)
Amhara	Andualem et al. [15]	2006	Gondar	Adult	Health facility	312	8.6	Stool	C$B	7
	Huruy et al. [16]	2008	Gondar	All	Health facility	384	16.9	Stool	C$B	7
	Andargie et al. [17]	2008	Gondar	Adult	Community	127	3.1	Stool	C$B	5
	Tiruneh [18]	2009	Gondar	All	Health facility	1200	7.5	Stool	C,B&S	5
	Huruy et al. [19]	2011	Gondar	All	Health facility	384	15.6	Stool	C$B	7
	Debas et al. [20]	2011	Bahir Dar	All	Health facility	215	14.9	Stool	C$B	7
	Demissie [21]	2014	Gondar	All	Health facility	372	4.6	Stool	C,B&S	9
	Abera et al. [22]	2016	Bahir Dar	Adult	Community	410	1.2	Stool	C$B	8
	Mulu et al. [23]	2017	DebreMarkos	All	Health facility	58	17.2	Stool	C$B	5
	Mengist et al. [24]	2018	DebreMarkos	Adult	Community	220	2.3	Stool	C$B	6
	Feleke et al. [25]	2018	Gonder	< 5 children	Health facility	225	2.2	Stool	C$B	9
Oromia	Mache [26]	2001	Jimma	Children	Health facility	384	20.1	Stool	C,B&S	7
	Beyene and Tasew [27]	2014	Jimma	Children	Health facility	260	2.3	Stool	C&B	7
	Surafel et al. [28]	2015	Ambo	All	Health facility	24	29	Stool	C$B	5
	Lamboro et al. [29]	2016	Jimma	All	Health facility	176	1.1	Stool	C$B	6
	Terfassa et al. [30]	2018	Nekemte	All	Health facility	422	2.1	Stool	C$B	8
	Marami et al. [31]	2018	Haramaya	Adult	Community	417	1.4	Stool	C$B	7
Southern	Roma et al. [32]	2000	Hawassa	All	Health Facility	289	34.6	Stool	C,B&S	5
	Mengistu et al. [33]	2014	Buta-jira	All	Health facility	382	4.5	Stool	C,B&S	6
	Mulatu et al. [34]	2014	Hawassa	<5 children	Health facility	158	7.0	Stool	C,B&S	8
	Mama and Alemu [35]	2016	Arba Minch	Adult	Community	345	3.0	Stool	C,B&S	9
	Ameya et al. [36]	2018	Arba Minch	<5 children	Health facility	167	4.8	Stool	C$B	9
	Abebe et al. [37]	2018	Hosanna	<5 children	Health facility	204	8.3	Stool	C$B	8
Central Ethiopia	Aklilu et al. [3﻿8﻿]	2015	Addis Ababa	Adult	Community	172	0.0	Stool	C$B	8
	Mamuye et al. [39]	2015	Addis Ababa	<5 children	Health facility	253	9.1	Stool	C$B	9
Hareri	Reda et al. [40]	2011	Harar	All	Health facility	244	6.7	Stool	C$B	6
Tigray	Gebrekidan et al. [41]	2015	Mekelle	All	Health facility	216	6.9	Stool	C$B	7
	Kahsay et al. [42]	2015	Mekelle	< 5 children	Health facility	241	13.3	Stool	C$B	9
	Gebreegziabher et al. [﻿43﻿]	2018	Mekelle	Children	Health facility	260	6.9	Stool	C$B	9

*Ref* reference, *PUB* publication, *C* culture, *B* biochemical, *S* serological

### Meta-analysis of *Shigella* species

**Fig. 3:**
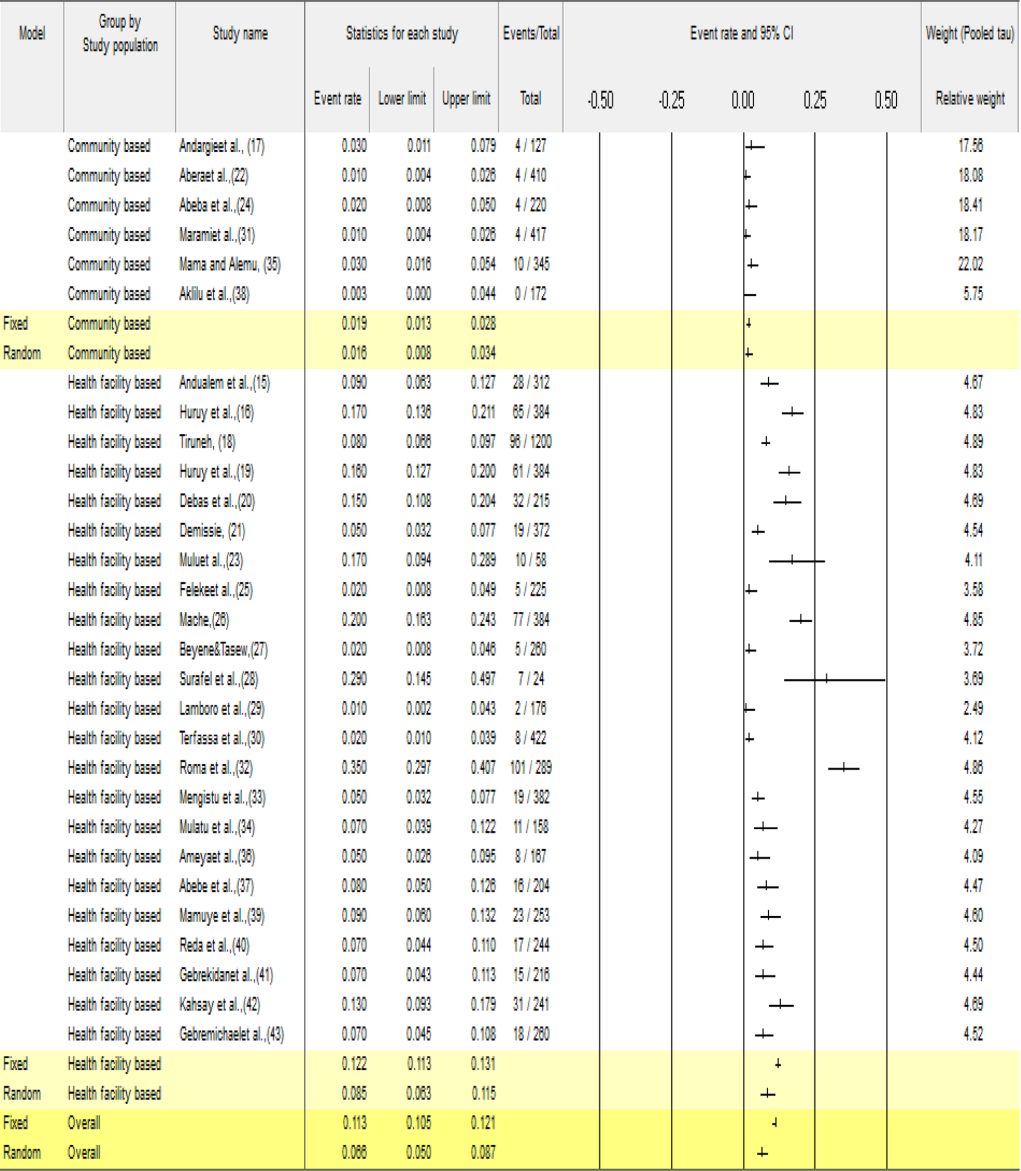
Forest plot for the prevalence of *Shigella* species in Ethiopia

The analysis of 29 studies, according to the Der Simonian–Laird random-effects model, revealed that the pooled prevalence of *Shigella* species in Ethiopia was 6.6% (95% CI 4.7–8.8) (Fig. 3). Pooled prevalence of *Shigella* species among regions was 7.0% (95% CI 4.6–10.3) in Amhara, 4.1% (95% CI 1.0–14.5) in Oromia, 7.7% (95% CI 2.7–20.1) in Southern Ethiopia, 2.2% (95% CI 0.1–40.5) in Addis Ababa, 7.0% (95% CI 4.4–11.0) in Hareri and 8.8% (95% CI 5.6–13.6) in Tigray. In year of study 26.9% (95% CI 14.8–43.8) in 1999–2003, 12.6% (95% CI 6.6–22.7) in 2004–2008, 8.1% (95% CI 5.1–12.8) in 2009–2013 and 4.9% (95% CI 3.3–7.0) from 2014 to 2018 (Additional file 1: Table S2) and among study population 1.8 (95% CI 1.1–2.9) were in health facility based and 1.6% (95% CI 0.8–3.4) in Community based studies. Among the above study parameters considered during meta-regression analysis, study years were associated with a decreasing *Shigella* prevalence over time (p = 0.0028).

### Meta-analysis of drug resistant *Shigella* species

The pooled resistant of *Shigella* species were 83.1% (95% CI 75.7–88.6) for ampicillin, 84.1% (95% CI 75.6–90.1) for Amoxicillin and 86.5% (95% CI 70.9–94.4) for erythromycin. Comparatively low resistance pattern was reported in ciprofloxacin 8.9% (95% CI 6.0–12.8), ceftriaxone 9.3% (95% CI 3.9–20.5), gentamycin 17.3% (95% CI 11.2–25.9), and norfloxacin 8.2% (95% CI 3.8–16.6). However intermediate resistance were recorded for augmentin (Amox + clav) 59.1% (95% CI 19.6–89.5) and co-trimoxazele 59.4% (95% CI 49.3–68.8). Moreover, resistant to more than one drugs or MDR were reported 83.2% (95% CI 77.1–87.9) (Table 2). Above 50% of *Shigella* developed resistance to ampicillin, erythromycin, tetracycline, cotrimoxazole and chloramphenicol which are the commonly prescribed antimicrobial drugs. Most studies on drug resistance were carried out in 2014–2018, though it is difficult to sufficiently address and compare the resistance pattern of commonly prescribed drugs through time (Additional file 1: Table S4).

**Table 2:** Pooled proportions of drug resistant *Shigella* isolated in Ethiopia from 1999 to 2018

	Antibiotic resistance rate reported by 25 studies												
	Roma et al. [32]	Mache [26]	Andualem et al. [15]	Huruy et al. [16]	Tiruneh et al. [18]	Huruy et al. [19]	Debas et al. [20]	Reda et al. [40]	Beyene and Tasew [27]	Demissie et al. [21]	Mengistu et al. [33]	Mulatu et al. [34]	Surafel et al. [28]
AMP	93	70.1	100	81.5	78.9	80	93.8	100	100	94.1	47.1	63.6	100
AMX	–	–	–	–	–	–	75	100	100	88.2	–	100	–
AMC	–	–	–	–	–	–	–	–	–	–	–	–	–
CEF	39	57.1	–	–	–	–	90.6	–	–	–	–	–	–
CHL	63	40.3	62	50.8	67.8	48.3	53.1	29.4	16.7	17.6	29.4	9.1	71.5
CIP	–	–	28	9.2	2.2	8.3	0	–	0	0	5.9	0	14.3
CRO	–	–	–	–	0	–	–	–	0	–	0	–	–
SXT	56	32.5	45	75.4	84.6	76.7	62.5	–	100	58.8	76.5	0	85.7
ERY	90	–	–	–	–	–	–	–	–	–	–	90.9	–
GEN	2	1.3	3	10.7	12.2	10	18.8	0	0	41.2	17.6	27.3	71.5
KAN	8	13	7	–	–	–	–	–	–	41.2	–	–	–
NAL	10	6.5	21	–	0	–	–	–	16.7	29.4	5.9	0	0
NOR	–	–	0	–	1.1	–	9.4	5.9	–	0	–	–	0
TET	90	–	86	87.7	90	85	93.8	70.6	–	88.2	82.4	–	71.5
MDR	82	85.7	96.6	81.5	94.5	83.3	93.8	100	100	94.1	50	100	85.7

	Antibiotic resistance rate reported by 25 studies												Pooled prevalence (95% CI)
	Gebrekidan et al. [41]	Mamuye et al. [39]	Mama and Alemu [35]	Lamboro et al. [29]	Mulu et al. [23]	Gebreegziabher et al. [43]	Ameya et al. [36]	Marami et al. [31]	Terfassa et al. [30]	Feleke et al. [25]	Abebe et al. [37]	Mengist [24]	
AMP	100	95.7	–	100	100	88.9	100	33.3	–	100	82.4	100	83.1 (75.7–88.6)
AMX	86.7		100		–	–	–	–	77.8	100	–	–	84.1 (75.6–90.1)
AMC	33.3	91.4	40	–	–	–	–	–	–	–	–	–	59.1 (19.6–89.5)
CEF	–	–	–	–	–	–	–	–	–	–	–	–	63.0 (37.3–83.0)
CHL	46.7	21.7	0	0	100	55.6	50	50	11.1	40	47.1	80	47.6 (39.9–55.5)
CIP	6.7	4.3		0	0	0	0	0	0	–	17.6	0	8.9 (6.0–12.8)
CRO	–	4.3	0	–	50	0	–	16.7	0	0	17.6		9.3 (3.9–20.05)
SXT	66.7	52.5	0	50	100	55.6	25	66.7	–	40	64.7	20	59.4 (49.3–68.8)
ERY	–	–	–	–	100	–	62.5	–	–	–	–	–	86.5 (70.9–94.4)
GEN	13.7	17.4	0	0	–	27.8	25	33.3	11.1	60	76.5	0	17.3 (11.2–25.9)
KAN	–	–	0	–	–	–	–	–	–	–	0	–	11.6 (5.9–21.6)
NAL	–	21.7	–	50		27.8	–	–	11.1	–	0	–	13.4 (8.4–20.6)
NOR	6.7	–	–	0	100	0	12.5	16.7	0	–	0	20	8.2 (3.8–16.6)
TET	–	–	–	100	100	77.8	–	83.3	–	60	–	80	86.1 (82.5–89.6)
MDR	80	87	100	100	60	88.9	75	85.7	33.3	100	63.2	NR	83.2 (77.1–87.9)

*AMP* ampicillin, *AMX* amoxicillin, *AMC* amoxicillin-clavulanic acid, *CEF* cephalothin, *CHL* chloramphenicol, *CIP* ciprofloxacin, *CRO* ceftriaxone, *SXT* trimethoprim–sulfamethoxazole, *ERY* erythromycin, *GEN* gentamicin, *KAN* kanamycin, *NAL* nalidixic acid, *NOR* norfloxacin, *TET* tetracycline, *MDR* multiple drug resistance

## Discussion

Antimicrobial resistance (AMR) is widely growing public health threat worldwide, particularly in resource limited countries including Ethiopia, where infectious diseases are widespread. It is now occurring across the world [1].

*Shigella* species is highly antibiotic resistant among eight drugs resistance bacteria. Regarding antibiotic resistance, if nothing is done, a person will die every three second by 2050 [44].

This meta-analysis determined the pooled prevalence of *shigella* species *in* Ethiopia using 29 studies. According to the results of this meta-analysis, the pooled prevalence was 6.6% (95% CI 4.7–8.8). This finding is in agreement with 6.6% (95% CI 3.4–9.7) *Shegilla* prevalence in systematic review among US military and similar populations [45].

Regional prevalence of *Shigella* species among regions was also calculated, hence a higher prevalence of *Shigella* species (8.8%) was reported in Tigray, which was nearly four times higher than the finding from Central Ethiopia (2.2%), even though the studies conducted and included in this review and meta-analysis from this region were small. The variations in prevalence estimates may be due to differences in the study populations and year of study.

Pooled prevalence of *Shigella* species by study year (26.9%) was reported in 1999.2003 and (4.9%) in 2014–2018. This finding is inconsistent with the study done in China [46]. This result showed a decreasing *Shigella* prevalence over time. The decreased in prevalence through time might be due to decrease in poverty, increase quality of life, rise of awareness on sanitation and hygiene, prevention and control strategy of communicable disease through deployed of health extension workers at community level across the country.

In this study, the pooled prevalence of shigellosis in children was (7.0%), while in adult population (2.2%). This finding confirms that *Shigella* cause diarrhoeal morbidity among infants and young children than adults [47] and the third leading cause of diarrhoeal deaths in children younger than 5 years [48]. The higher occurrence of *Shigella* in children compared to adults suggests a higher vulnerability of children to *Shigella* infection, this may be due to the unhygienic food handling practices, compromised sanitation, malnutrition and the ability to cause disease with a small inoculum and facilitating spread from person to person [49].

Based on the data obtained from twenty-five published articles; *Shigella* species showed high resistance to amoxicillin, ampicillin, and erythromycin. This finding is in line with the study done in Germany [50].

In Ethiopia the drug of choice on shigellosis treatment is norfloxacin, ciprofloxacin, and ceftriaxone for adult. This is similar with guideline USA [51]. However this review and meta-analysis showed slightly high resistance was reported on norfloxacin, ciprofloxacin, and ceftriaxone.

Furthermore, the occurrences of *Shigella* isolates resistant to two or more drugs were high (83.2%). This increment may be due mobile genetic units (including plasmids, gene cassettes in integrons and transposons) [1], inadequate access to effective drugs, unregulated dispensing, truncated antimicrobial therapy, medication sharing, counterfeit drugs, bacterial evolution, climate changes, lack of medical practitioner with proper training, poor-quality and unhygienic sanitary conditions [52–54].

In this review, included studies primarily used stool culture for *Shigella* identification. This estimate appears to be less sensitive method than molecular methods and may underestimate the actual occurrence of this bacteria [47].

## Conclusion

The pooled estimate provides high burden of *Shigella* infection and its high proportion of drug resistance pattern to ampicillin, Amoxicillin and Erythromycin in Ethiopia. Therefore, initiating and scale up of performing drug susceptibility test for each shigellosis case, educate the community and health care providers on appropriate use of antibiotics need to be considered and strengthened.

## Limitation

The patient data were collected from urban and rural populations of the country that took service from hospitals but urban dwellers have better access to health care setting. Therefore, the pooled estimates are more applicable to patients in urban areas than rural. Resistance pattern of shigellosis was not reported for money drugs by some studies and in most studies *Shigella* infection were not tested at species level.

## Additional files

**Additional file 1: Table S1.** Study design and quality assessment of the studies included in systematic review and meta-analysis of *shigella species.* **Table S2.** Subgroup meta-analysis of *Shigella* species prevalence estimation in Ethiopia from 1999 to 2018. **Table S3:** Microbiological methods used to isolate and identify *Shigella* species in Ethiopia from 1999 to 2018. **Table S4.** Prevalence of drug resistance (95% CI) for *Shigella* from 1999 to 2018.

**Additional file 2: Figure S1.** Egger regression intercept of analysis of included studies reporting on the prevalence of *Shigella* species in Ethiopia. **Figure S2.** Begg and Mazumdar rank correlation of analysis of included studies reporting on the prevalence of *Shigella* species in Ethiopia.

## Data Availability

There is no remaining data and materials, all information is clearly presented in the main manuscript.

## Abbreviations

AMR: antimicrobial resistance
MDR: multi-drug resistant
