# Supplementary material for: Prevalence of Shigella species and its drug resistance pattern in Ethiopia: a systematic review and meta-analysis
Source: Ann Clin Microbiol Antimicrob. 2019 Jul 9;18:22. doi: 10.1186/s12941-019-0321-1 (PMC6617577; doi:10.1186/s12941-019-0321-1)
Supplement: Supplementary file 1 — Additional file 1: Table S1. Study design and quality assessment of the studies included in systematic review and meta-analysis of shigella species. Table S2. Subgroup meta-analysis of Shigella species prevalence estimation in Ethiopia from 1999 to 2018. Table S3: Microbiological methods used to isolate and identify Shigella species in Ethiopia from 1999 to 2018. Table S4. Prevalence of drug resistance (95% CI) for Shigella from 1999 to 2018. [file 12941_2019_321_MOESM1_ESM.docx]

Table S1: Study design and quality assessment of the studies included in systematic review and meta-analysis of *shigella species*

| **Publication** | Study design | 1 | 2 | 3 | 4 | 5 | 6 | 7 | 8 | 9 | **Quality score (9%)** |
| --- | --- | --- | --- | --- | --- | --- | --- | --- | --- | --- | --- |
| Andualem et al.,(15) | CS | Y | NR | Y | Y | Y | Y | Y | NA | Y | 7 |
| Huruy et al.,(16) | CS | Y | Y | Y | Y | Y | Y | NR | NA | Y | 7 |
| Andargie*et al.,* (17) | CS | NA | Y | NR | Y | Y | Y | NR | NA | Y | 5 |
| Tiruneh, (18) | CS | Y | Y | NR | Y | Y | Y | NR | NA | NR | 5 |
| Huruy et al.,(19) | CS | Y | Y | NR | Y | Y | Y | NR | Y | Y | 7 |
| Debas et al.,(20) | CS | Y | Y | NR | Y | Y | Y | NR | Y | Y | 7 |
| Demissie, (21) | CS | Y | Y | Y | Y | Y | Y | Y | Y | Y | 9 |
| Abera*et al.,(22)* | CS | NA | Y | Y | Y | Y | Y | Y | Y | Y | 8 |
| Mulu*et al.,(23)* | CS | NR | Y | NA | Y | Y | Y | NA | Y | NR | 5 |
| Abeba et al.,(24) | CS | NA | Y | NR | Y | Y | Y | NR | Y | Y | 6 |
| Felekeet al.,(25) | CS | Y | Y | Y | Y | Y | Y | Y | Y | Y | 9 |
| Mache,(26) | CS | Y | Y | Y | Y | Y | Y | NR | N | Y | 7 |
| Beyene&Tasew,(27) | CS | Y | Y | Y | Y | Y | Y | NR | NO | Y | 7 |
| Surafel et al.,(28) | CS | N | Y | y | Y | Y | Y | NR | N | NR | 5 |
| Lamboro et al.,(29) | CS | Y | Y | N | Y | Y | Y | NR | N | Y | 6 |
| Terfassa et al.,(30) | CS | Y | Y | Y | Y | Y | Y | Y | NO | y | 8 |
| Marami*et al.,(31)* | CS | NA | Y | Y | Y | Y | Y | Y | NO | Y | 7 |
| Roma et al.,(32) | CS | Y | Y | NR | Y | Y | Y | NR | N | NA | 5 |
| Mengistu et al.,(33) | CS | Y | Y | NR | Y | Y | Y | Y | N | NR | 6 |
| Mulatu et al.,(34) | CS | Y | Y | Y | Y | Y | Y | NR | Y | Y | 8 |
| Mama and Alemu, (35) | CS | Y | Y | Y | Y | Y | Y | Y | Y | Y | 9 |
| Ameya*et al.,(36)* | CS | Y | Y | Y | Y | Y | Y | Y | Y | Y | 9 |
| Abebe et al.,(37) | CS | Y | Y | Y | Y | Y | Y | Y | N | Y | 8 |
| Aklilu et al.,(38) | CS | NA | Y | Y | Y | Y | Y | Y | Y | Y | 8 |
| Mamuye et al.,(39) | CS | Y | Y | Y | Y | Y | Y | Y | Y | Y | 9 |
| Reda et al.,(40) | CS | Y | N | Y | Y | Y | Y | NR | N | Y | 6 |
| Gebrekidan*et al.,* (41) | CS | Y | NR | Y | Y | Y | Y | NR | Y | Y | 7 |
| Kahsay et al.,(42) | CS | Y | Y | Y | Y | Y | Y | Y | Y | Y | 9 |
| Gebremichael*et al*.,(43) | CS | Y | Y | Y | Y | Y | Y | Y | Y | Y | 9 |

**Key:** **Y**= Yes; **N**=No; **NR**= Not reported

**Question codes:**

1. Wasthesampleframeappropriatetoaddressthetarget population?

2. Werestudyparticipantssampledinanappropriate way?

3. Wasthesamplesize adequate?

4. Werethestudysubjectsandthesettingdescribedin detail?

5. Wasthedataanalysisconductedwithsufficientcoverageoftheidentified sample?

6. Werevalidmethodsusedfortheidentificationofthecondition?

7. Was the condition measured in a standard, reliable way for all participants?

8. Wasthereappropriatestatisticalanalysis?

9.Was the response rate adequate, and if not, was the low response rate managed appropriately?

Table S2: Subgroup meta-analysis of Shigella species prevalence estimation in Ethiopia from 1999-2018

| Categories | Subgroup | Studies included | Prevalence %  (95% CI) | I^2^% | P-v |
| --- | --- | --- | --- | --- | --- |
| Region | Amhara | 11 | 7.0(4.6-10.3) | 91.2 | <0.0001 |
|  | Oromia | 6 | 4.1(1.0-14.5) | 95.71 | <0.0001 |
|  | Southern Ethiopia | 6 | 7.7(2.7-20.1) | 96.97 | <0.0001 |
|  | Central Ethiopia (A.A) | 2 | 2.2(0.1-40.5) | 83.56 | =0.014 |
|  | Hareri | 1 | 7.0(4.4-11.0) | 0.000 | 1.00 |
|  | Tigray | 3 | 8.8(5.6-13.6) | 70.67 | =0.033 |
| Year of study | 1999-2003 | 2 | 26.9(14.8-43.8) | 94.65 | <0.0001 |
|  | 2004-2008 | 2 | 12.6(6.6-22.7) | 89.13 | <0.002 |
|  | 2009-2013 | 4 | 8.1(5.1-12.8) | 82.04 | =0.001 |
|  | 2014-2018 | 21 | 4.9(3.3-7.0) | 88.70 | <0.0001 |
| Age group | Children | 9 | 7.0(4.4-11.2) | 89.69 | <0.0001 |
|  | Adult | 7 | 2.2(0.9-4.9) | 85.86 | <0.0001 |
|  | All age group | 13 | 9.6(6.1-14.8) | 95.15 | <0.0001 |
| Study population | Community based | 6 | 1.6(0.8-3.4) | 39.64 | 0.141 |
|  | Health facility based | 23 | 8.5(6.2-11.5) | 93.37 | <0.001 |

**Table S3:** Microbiological methods used to isolate and identify Shigella species in Ethiopia from 1999 - 2018.

| **First Author** | **Media** | **Biochemical test** | **Sero-group** |
| --- | --- | --- | --- |
| Andualem et al.,(15) | MA | TSIA, SCA, UT, LIA, MB, GB and OT | NR |
| Huruy et al.,(16) | MA | TSIA, SCA, IT, , LD, UT and MA | NR |
| Andargie*et al.,* (17) | SS, MA | NR | NR |
| Tiruneh, (18) | SS, MA | TSIA, SCA, MT,UT,LIA, IT, and MB | Yes |
| Huruy et al.,2011(19) | MA, SS | NR | NR |
| Debas et al.,(20) | MA, XLD,SFB | NR | NR |
| Demissie, (21) | SS, MA,SFB | TSIA, SCA, MT, UT, LIA and IT | Yes |
| Abera*et al.,(22)* | XLD.MA,SFB | NR | NR |
| Mulu*et al.,(23)* | XLD.MA | NR | NR |
| Abeba et al.,(24) | SFB , XLD | IT, MT, LD, TSI, SCA, and UT | NR |
| Felekeet al.,(25) | XLD,DCA | NR | NR |
| Mache,(26) | MA, SS,SFB | NR | NR |
| Beyene&Tasew,(27) | DCA, XLD,SFB | NR | NR |
| Surafel et al.,(28) | MA, SS, XLD,SFB | TSIA,MT, UT, LIA and MB | NR |
| Lamboro et al.,(29) | XLD,SFB | NR | Yes |
| Terfassa et al.,(30) | SS,XLD | MT,LIA,KIA,SCA, and IT | NR |
| Marami*et al.,(31)* | SFB,XLD | MT,IT and KIA | NR |
| Roma et al.,(32) | MA, XLD,SS | KIA, SCA, MT, UT, LIA, OT and IT | Yes |
| Mengistu et al.,(33) | MA,XLD,SS ,SFB | API 20E | Yes |
| Mulatu et al.,(34) | DCA, XLD,SFB | NR | Yes |
| Mama and Alemu, (35) | MA,XLD | KIA, SCA UT, LIA and IT | NR |
| Ameya*et al.,(36)* | MA,XLD | NR | NR |
| Abebe et al.,(37) | SFB,DCA,XLD | NR | NR |
| Aklilu et al.,(38) | XLD,SFB | NR | NR |
| Mamuye et al.,(39) | MA,DCA,SS,SFB | NR | NR |
| Reda et al.,(40) | DCA,XLD | KIA, MT, UT and IT | NR |
| Gebrekidan*et al.,* (41) | MA,XLD,DCA,SFB | KIA, SCA, MT, UT LIA, and IT | NR |
| Kahsay et al.,(42) | MA,XLD | KIA,MT,IT,LIA,SCA and UT | NR |
| Gebremichael*et al*.,(43) | MA,XLD,SFB | API 20E | Yes |

**Keys: XLD**: Xylose Lysine Desoxycholate agar, **MA**:MacConkey, **DCA**:Deoxycholate Citrate Agar, **SS**:Salomonella, Shigella Agar, **SFB**: Selenite F broth **TSI:**Triple Sugar Iron Agar, **KIA**:Kligler iron agar ,**SCA**:Simon’s Citrate Agar, **LIA**:Lysine Iron Agar, **MT**:Motility, **MB**:Mannitol Broth, **GB**:Glucose Broth, **LD**:Lysine Decarboxylase, **UT**:Urease Test, **IT**:Indole Test, **API**:Analytical Profile Index,**OT**:Oxidase test, **Nr:**not reported

Table S4: Prevalence of drug resistance (95% CI) for Shigella from 1999-2018

| **Antimicrobial agents** | **Classification of drug desistance** | **No. of studies** | **Prevalence of drug resistance (95% CI) (%)** | **I^2^** | **P-value** |
| --- | --- | --- | --- | --- | --- |
|  | **Stratified by years** |  |  |  |  |
| Ampicillin | 1999-2003 | 2 | 84.5(49.8-96.7) | 92.836 | <0.0001 |
|  | 2004-2008 | 2 | 91.4(50.1-99.1) | 66.45 | 0.084 |
|  | 2009-2013 | 4 | 83.8(73.6-90.6) | 44.58 | 0.144 |
|  | 2014-2018 | 15 | 84.5(72.4-91.9) | 51.91 | 0.010 |
| Amoxicillin | 2009-2013 | 2 | 87.5(41.5-98.6) | 63.16 | 0.099 |
|  | 2014-2018 | 8 | 88.5(78.9-94.1) | 0.00 | 0.941 |
| Amoxc+clavul. | 2014-2018 | 3 | 60.6(18.4-91.3) | 97.90 | <0.0001 |
| Cephalothin | 1999-2003 | 2 | 48.0(31.1-65.5) | 95.52 | <0.0001 |
|  | 2009-2013 | 1 | 90.6(87.2-93.2) | 0.00 | 1.000 |
| Chloramphenicol | 1999-2003 | 2 | 51.7(30.2-72.6) | 97.13 | <0.0001 |
|  | 2004-2008 | 2 | 55.7(44.6-66.2) | 82.44 | 0.017 |
|  | 2009-2013 | 4 | 49.5(33.1-66.0) | 97.36 | <0.0001 |
|  | 2014-2018 | 17 | 33.6(22.9-46.4) | 97.26 | <0.0001 |
| Ciprofloxacin | 1999-2003 | 2 | 16.6(5.0-42.6) | 79.66 | 0.027 |
|  | 2004-2008 | 3 | 4.2(1.4-11.9) | 42.51 | 0.176 |
|  | 2009-2013 | 15 | 7.8(0.5-62.2) | 0.0 | 0.574 |
| Ceftriaxone | 2009-2013 | 1 | 0.5(0.0-8.2) | 0.0 | 1.000 |
|  | 2014-2018 | 10 | 12.5(5.9-24.6) | 31.89 | 0.153 |
| Trimethoprim-sulfamethoxazole | 1999-2003 | 2 | 43.9(23.2-67.0) | 97.37 | <0.0001 |
|  | 2004-2008 | 2 | 61.5(30.5-85.4) | 95.92 | <0.0001 |
|  | 2009-2013 | 3 | 75.6(59.6-85.7) | 95.75 | <0.0001 |
|  | 2014-2018 | 16 | 53.9(42.4-65.1) | 96.24 | <0.0001 |
| Erythromycin | 1999-2003 | 1 | 90.0(82.4-94.5) | 0.0 | 1.000 |
|  | 2014-2018 | 3 | 83.7(52.2-96.0) | 43.04 | 0.173 |
| Gentamicin | 1999-2003 | 2 | 1.7(0.6-5.2) | 0.0 | 0.723 |
|  | 2004-2008 | 2 | 8.4(3.1-20.9) | 21.83 | 0.258 |
|  | 2009-2013 | 4 | 12.5(8.5-18.0) | 0.00 | 0.432 |
|  | 2014-2018 | 16 | 28.8(18.8-41.6) | 51.79 | 0.008 |
| Kanamycin | 1999-2003 | 2 | 10.4(6.4-16.5) | 14.47 | 0.280 |
|  | 2014-2018 | 3 | 11.4(1.4-53.6) | 60.73 | 0.078 |
| Nalidixic acid | 1999-2003 | 2 | 8.6(5.3-13.9) | 0.0 | 0.411 |
|  | 2004-2008 | 1 | 21.0(9.5-40.2) | 0.0 | 1.00 |
|  | 2009-2013 | 1 | 0.5(0.0-8.2) | 0.0 | 1.00 |
|  | 2014-2018 | 11 | 19.4(12.9-28.2) | 0.0 | 0.451 |
| Norfloxacin | 2004-2008 | 1 | 1.8(0.1-23.0) | 0.0 | 1.000 |
|  | 2009-2013 | 2 | 3.8(0.4-25.6) | 72.16 | 0.058 |
|  | 2014-2018 | 12 | 11.1(4.6-24.2) | 36.982 | 0.095 |
| Tetracycline | 1999-2003 | 1 | 90.0(82.4-94.5) | 0.0 | 1.000 |
|  | 2004-2008 | 2 | 87.2(78.7-92.6) | 0.0 | 0.824 |
|  | 2009-2013 | 4 | 86.3(77.0-92.2) | 48.47 | 0.121 |
|  | 2014-2018 | 9 | 80.4(70.2-87.7) | 0.0 | 0.888 |
| Multiple drug resistance | 1999-2003 | 2 | 83.5(77.3-88.3) | 0.0 | 0.511 |
|  | 2004-2008 | 2 | 89.5(59.7-98.0) | 64.61 | 0.093 |
|  | 2009-2013 | 4 | 91.6(82.4-96.2) | 52.05 | 0.100 |
|  | 2014-2018 | 17 | 77.7(67.3-85.5) | 39.86 | 0.046 |
