# Supplementary material for: Prevalence of Shigella species and its drug resistance pattern in Ethiopia: a systematic review and meta-analysis
Source: Ann Clin Microbiol Antimicrob. 2019 Jul 9;18:22. doi: 10.1186/s12941-019-0321-1 (PMC6617577; doi:10.1186/s12941-019-0321-1)
Supplement: Supplementary file 2 — Additional file 2: Figure S1. Egger regression intercept of analysis of included studies reporting on the prevalence of Shigella species in Ethiopia. Figure S2. Begg and Mazumdar rank correlation of analysis of included studies reporting on the prevalence of Shigella species in Ethiopia. [file 12941_2019_321_MOESM2_ESM.docx]

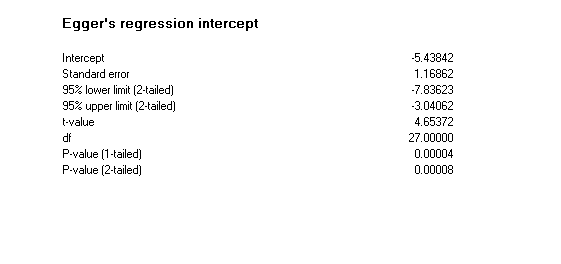


Figure S1: Egger regression intercept for the prevalence of Shigella species in Ethiopia


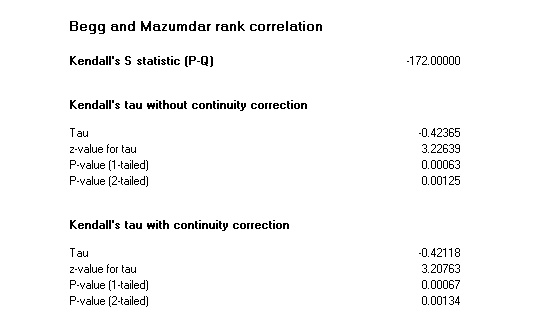


Figure S2: Begg and mazumdar rank correlation of analysis for the prevalence of Shigella species in Ethiopia
